# Preoperative circulating tumor cells integrated with imaging analysis for prognostic evaluation in head and neck squamous cell carcinoma

**DOI:** 10.1002/1878-0261.70299

**Published:** 2026-07-04

**Authors:** Susanne Flach, Gizem Abaci, Tom Huberty, Axel Lechner, Lukas Käsmann, Jens Ricke, Sophia Stöcklein, Martin Canis, Philipp Baumeister, Marianna Alunni‐Fabbroni

**Affiliations:** ^1^ Department of Otorhinolaryngology, Head and Neck Surgery LMU University Hospital, LMU Munich Germany; ^2^ German Cancer Consortium (DKTK), Partner Site Munich Germany; ^3^ Department of Radiology LMU University Hospital, LMU Munich Germany; ^4^ Department of Radiotherapy and Radiation Oncology LMU University Hospital, LMU Munich Germany; ^5^ Department of Radiation Oncology Klinikum Landshut Germany; ^6^ European Liquid Biopsy Society (ELBS) Hamburg Germany

**Keywords:** circulating tumor cells, head and neck squamous cell carcinoma, imaging analysis, liquid biopsy, surgery

## Abstract

Head and neck squamous cell carcinoma (HNSCC) is associated with high locoregional recurrence and poor survival despite multimodal treatment. Reliable biomarkers to guide personalized therapy remain lacking. Circulating tumor cells (CTCs) represent a promising tool, but their clinical utility in HNSCC is insufficiently defined. In this cohort study, 30 stage I–IVb HNSCC patients undergoing curative‐intent surgery were enrolled. Preoperative blood samples were analyzed using the FDA‐approved CellSearch™ system for CTC count and Programmed death‐ligand 1 (PD‐L1) expression. Prognostic associations were assessed using Kaplan–Meier analysis and Cox regression. Combined risk models integrating CTC status with radiological features were explored. CTCs were detected in 37.9% (11/29) of patients. CTC positivity was significantly associated with reduced overall survival (*P* = 0.031). Univariate and bootstrap‐adjusted Cox regression identified CTC presence and tumor volume as factors associated with overall survival. Integrating CTC status with tumor volume or nodal stage improved patient stratification, identifying a high‐risk group with shorter recurrence‐free and overall survival. Preoperative CTC detection may serve as a prognostic biomarker in HNSCC and enhances risk stratification when combined with imaging‐derived tumor characteristics, supporting liquid biopsy integration into pre‐surgical evaluation.

## Graphical abstract

Detecting circulating tumor cells (CTCs) in blood before surgery may help predict outcomes in patients with head and neck squamous cell carcinoma (HNSCC). Here, we show when combined with tumor size and lymph node involvement from routine imaging, CTC status identifies high‐risk patients with poorer survival—offering a simple, minimally invasive tool to guide more personalized treatment decisions. Figure generated with biorender.

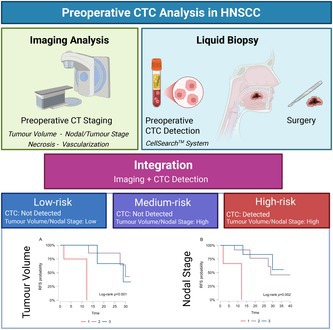

**Abbreviations**
CT: Computed tomography
CTC: Circulating tumor cell
ctDNA: circulating cell‐free tumor DNA
DAPI: 4′,6‐diamidino‐2‐phenylindole dihydrochloride
EGFR: Epidermal growth factor receptor
EMT: Epithelial–mesenchymal transition
EpCAM: Epithelial cell adhesion molecule
FDA: Food and drug administration
FITC: Fluorescein isothiocyanate
HNSCC: Head and neck squamous cell carcinoma
HR: Hazard ratio
LMU: Ludwig‐Maximilians‐Universität
MRI: Magnetic resonance imaging
OS: Overall survival
PD‐L1: Programmed death‐ligand 1
RFS: Recurrence‐free survival
RT‐PCR: Reverse transcription polymerase chain reaction

## 1 Introduction

Squamous cell carcinomas of the upper aerodigestive tract (head and neck squamous cell carcinoma, HNSCC) are among the seventh most common malignant diseases worldwide [1, 2]. Despite improved treatment options, up to 30% of patients with HNSCC develop local recurrences and/or a second primary tumor, resulting in a 5‐year survival rate that remains below 50–60% [3, 4, 5, 6]. Despite advances in treatment strategies, effectively treating locally advanced tumors remains challenging and still necessitates a multimodal therapeutic approach. Treatment options include surgical resection of the primary tumor and regional lymph node metastases, chemo(radio)therapy, or a combination of treatment modalities [7]. Currently, biomarkers to provide personalized therapy planning do not exist for primary HNSCC. The use of liquid biopsies to detect circulating cell‐free tumor DNA (ctDNA) and circulating tumor cells (CTC) is becoming increasingly important in this context [8, 9]. These minimally invasive diagnostic methods involve isolating and analyzing ctDNA and CTC from bodily fluids such as blood or saliva [10, 11]. CTCs, shed from primary tumors into the blood and lymphatic circulation, act as pioneers of metastasis by seeding distant sites and uniquely enabling the capture and study of the most aggressive cancer clones to reveal insights into metastatic biology [12]. Among the various platforms for analyzing CTCs, the FDA‐approved CellSearch™ system (Menarini Silicon Biosystems, Italy) employs immunomagnetic selection based on epithelial cell adhesion molecule (EpCAM) expression [11], a marker commonly expressed on HNSCC cells, although its expression may vary depending on epithelial–mesenchymal transition (EMT) status and tumor differentiation [13, 14, 15]. Based on CellSearch™, CTC detection rate ranges from 13% to 40% [16, 17, 18, 19]. However, while CTC enumeration and molecular profiling have shown prognostic value in other solid malignancies [20, 21], their application in HNSCC is less established. Here, we aim to evaluate the role of preoperative CTC analysis in patients with HNSCC who received surgical treatment with curative intent, focusing on its potential to inform prognostication and risk stratification. By exploring the relationship between preoperative CTC counts, radiological characteristics, and clinical outcomes, this study seeks to elucidate the relevance of CTC analysis in the pre‐surgical setting and its implications for optimizing patient management in HNSCC.

## 2 Material and methods

### 2.1 Study design and patient cohort

We conducted a single‐center prospective cohort study, which has been approved by the local ethics committee of the LMU University Hospital in Munich (ref. no. 18‐446). All accrued patients signed written informed consent prior to enrollment. Study participants consented to sharing pseudonymized data and samples within and outside of the European Union. The study has been conducted in accordance with the Declaration of Helsinki and in keeping with the rules of good clinical practice and according to the German laws and ethical standards. Eligible patients had pathologically confirmed stage I–IVb [22] HNSCC of the oral cavity, pharynx, or larynx and received surgical treatment with curative intent. Patients with other active malignancies or distant metastasis at the time of enrollment were excluded. Patients were staged with computed tomography (CT) and/or magnetic resonance imaging (MRI). Thirty patients were enrolled into the study at the Department of Otorhinolaryngology, Head and Neck Surgery at the LMU University Hospital between February 2022 and August 2024. Patients received adjuvant chemo(radio)therapy according to the National Comprehensive Cancer Network guidelines [23] following discussion by a local multidisciplinary tumor board. Immunohistochemical staining for p16 was performed as part of the routine diagnostic work‐up. Patients were followed up clinically and by regular ultrasound imaging of the neck, clinical examination, and CT/MRI imaging, when indicated. Patient demographics were collected (Table 1), and study participants were followed up for recurrence‐free survival (RFS) and overall survival (OS). RFS is defined as the time from treatment start to disease recurrence or loss to follow‐up. OS is the time from start of treatment to death or loss to follow‐up. Recurrence was assessed using histopathological results from biopsies and imaging studies taken during follow‐up.

**Table 1:** Patient characteristics.

Variable	All (*n* = 29; 100%)	CTC positive (*n* = 11; 37.9%)	CTC negative (*n* = 18; 62.1%)	*P*
Age				1.00
Median (range)	64 (51–81)	69 (51–79)	64 (51–81)	
Gender				1.00
Female	3 (10.3)	1 (9.0)	2 (11.1)	
Male	26 (89.7)	10 (91.0)	16 (88.9)	
Smoking status				0.55
Active smoker	15 (51.7)	6 (54.5)	9 (50.0)	
Ex‐smoker	9 (31.0)	2 (18.2)	7 (38.9)	
Never‐smoker	5 (17.3)	3 (27.3)	2 (11.1)	
Drinking status				1.00
Active	22	8 (72.7)	14 (77.8)	
None	7	3 (27.3)	4 (22.2)	
Location				0.28
Oral cavity	6 (20.7)	3 (27.3)	3 (16.7)	
Oropharynx	6 (20.7)	2 (18.2)	4 (22.2)	
Larynx	8 (27.6)	1 (9.1)	7 (38.9)	
Hypopharynx	9 (31.0)	5 (45.4)	4 (22.2)	
Tumor volume (mm^3^)				1.00
Median (range)	5784.85 (160.00–27920.00)	5200.70 (1600.00–22236.82)	6369.85 (160.00–27920.00)	
Necrosis				0.69
Yes	20 (68.9)	7 (63.6)	13 (72.2)	
No	9 (31.1)	4 (36.4)	5 (27.7)	
Vascularization				0.93
Yes	22 (75.9)	8 (27.7)	14 (77.8)	
No	5 (17.2)	2 (18.2)	3 (16.7)	
N/A	2 (6.9)	1 (9.1)	1 (5.5)	
p16 status				1.00
Positive	5 (17.25)	2 (18.2)	3 (16.7)	
Negative	24 (82.75)	8 (72.8)	15 (83.3)	
pT stage				0.19
pT1	4 (13.8)	0 (0.0)	4 (22.2)	
pT2	13 (44.8)	5 (45.45)	8 (44.4)	
pT3	8 (27.6)	5 (45.45)	3 (16.7)	
pT4	4 (13.8)	1 (9.1)	3 (16.7)	
pN stage				0.63
pN0	11 (37.9)	5 (45.4)	6 (33.3)	
pN1	5 (17.2)	2 (18.2)	3 (16.7)	
pN2	4 (13.8)	1 (9.1)	3 (16.7)	
pN3	6 (20.7)	2 (18.2)	4 (22.2)	
pNx	1 (3.4)	1 (9.1)	0 (0.0)	
N/A	2 (6.9)	0 (0.0)	2 (11.1)	
Prognostic stage				0.71
I + II	14 (48.3)	6 (54.5)	8 (44.4)	
III + IV	15 (51.7)	5 (45.5)	10 (55.6)	

### 2.2 Isolation and enumeration of CTC

Blood samples (7.5 mL peripheral blood) were collected at baseline in CellSave™ preservative tubes (Menarini Silicon Biosystem, Bologna, Italy) and processed using the FDA‐cleared CellSearch™ System (Menarini Silicon Biosystem), including the CellSearch CTC Kit (Menarini Silicon Biosystem) for CTC enrichment and enumeration. This system is based on immunomagnetic capture and enrichment of EpCAM‐positive cells using antibody‐coated ferrofluids, followed by staining for epithelial markers (cytokeratin 8, 18, and 19) and the leukocyte marker CD45, combined with nuclear staining using the fluorescent dye 4′,6‐diamidino‐2‐phenylindole dihydrochloride (DAPI). For PD‐L1 assessment, an additional immunofluorescence staining step was performed using a fluorescein isothiocyanate (FITC)‐conjugated anti‐human PD‐L1 (B7‐H1) monoclonal antibody (R&D Systems, Minneapolis, USA) at a final concentration of 20 μg·mL^−1^, as previously described [21]. PD‐L1 expression was evaluated on identified CTCs based on fluorescence signal intensity within the FITC channel. Identification and enumeration of CTCs were conducted using a semi‐automated fluorescence imaging system, with independent review by two trained operators. Identification and enumeration of CTCs were conducted with a semi‐automated fluorescence‐based microscope system. Two trained users reviewed the image galleries independently. Samples in which at least one CTC was detected were classified as CTC‐positive, while samples lacking detectable CTCs were categorized as CTC‐negative.

### 2.3 Radiological imaging analysis

Preoperative radiological imaging was performed as part of routine staging using contrast‐enhanced CT of the head, neck, and chest. All scans were evaluated by board‐certified radiologists at the Department of Radiology, LMU University Hospital, according to the 8th edition TNM classification of head and neck cancer. Tumor segmentation and quantitative feature extraction were performed using the structured Mint Lesion™ platform (Mint Medical, Heidelberg, Germany), ensuring standardized volumetric and morphological assessment. The following imaging biomarkers were recorded: primary tumor volume (mm^3^) (pT1‐4), intratumoral necrosis (graded as absent, little, or extensive), vascularization (graded as low, moderate, or high), and nodal stage (cN0–cN3) based on radiological criteria. Regional lymph nodes were further evaluated for size, morphology, and presence of extranodal extension. Imaging‐derived features were subsequently integrated with CTC status for combined prognostic analyses.

### 2.4 Statistical analysis

Normality of the variables was assessed using the Shapiro–Wilk test. For comparisons between two groups, normally distributed variables were analyzed using paired or unpaired *t*‐tests, while non‐normally distributed variables were compared using the Mann–Whitney *U*‐test. For categorical variables with two possible outcomes, Fisher's exact test was used, while for categorical variables with more than two possible outcomes, chi‐square test was used. RFS and OS were estimated using the Kaplan–Meier method, and statistical significance was evaluated using the log‐rank test. Univariate Cox proportional hazards regression analysis was conducted to identify prognostic factors. To assess the stability of the identified associations, internal validation was performed using bootstrap resampling (1000 iterations). Bootstrap‐derived *P*‐values were used descriptively to evaluate the consistency of effect estimates rather than as primary measures of statistical significance. For all analysis, a two‐sided *P*‐value of < 0.05 was considered statistically significant. *P*‐values were marked as * < 0.05, ** < 0.01, *** < 0.001. Statistical analysis was performed with graphpad prism, version 10.2.3 (GraphPad, San Diego, CA, USA), jasp statistical software, version 0.19.3 (JASP Team), and ibm spss, version 29.0.

## 3 Results

### 3.1 Patient baseline characteristics

Thirty patients were enrolled between February 2022 and August 2024, but one patient was excluded from further analysis due to technical failure. Twenty‐nine patients (26 men, 3 women) with a median age of 64 years (range 51–81 years) received curative‐intent surgery. Half of the patients (15/29, 51.7%) had stage III/IV disease and 82.8% were diagnosed with p16‐negative HNSCC (24/29). Patients presented at diagnosis with hypopharyngeal cancer (9/29, 31.0%) or laryngeal cancer (8/29, 27.6%), followed by equal distribution of cancers of the oral cavity and oropharynx (each 6/29, 20.7%). The majority of patients (25/29, 86.2%) were active or former smokers with a median of 31 pack years. Adjuvant therapy was recommended by the local tumor board in 55.2% (16/29) of the cases and administered as chemoradiotherapy with concomitant cisplatin in 13/16 (81.3%) and radiotherapy alone in 3/16 (18.7%) of the cases. Out of 29 patients, seven patients (24.1%) experienced recurrences of disease, with 5/7 (71.4%) cases of local or regional recurrence and 2/7 (28.6%) cases with distant disease relapse. Patients' characteristics are summarized in Table 1.

### 3.2 CTC detection in preoperative blood samples

Of the 30 patients enrolled in the study, CTC assessment using CellSearch™ was successful in 96.7% (29/30) prior to surgery. One patient had incomplete CTC analysis due to poor sample quality and CellSearch™ failure of CTC detection. Baseline CTCs were detected in 37.9% (11/29) of patients, of whom three patients experienced recurrence of their disease (one local recurrence, one locoregional recurrence, and one distant recurrence). Median number of detected CTCs was one (range 1–5). PD‐L1 expression on CTCs was observed in 2 (18.2%) cases. Notably, none of these patients experienced disease relapse during the follow‐up period.

**Fig. 1:**
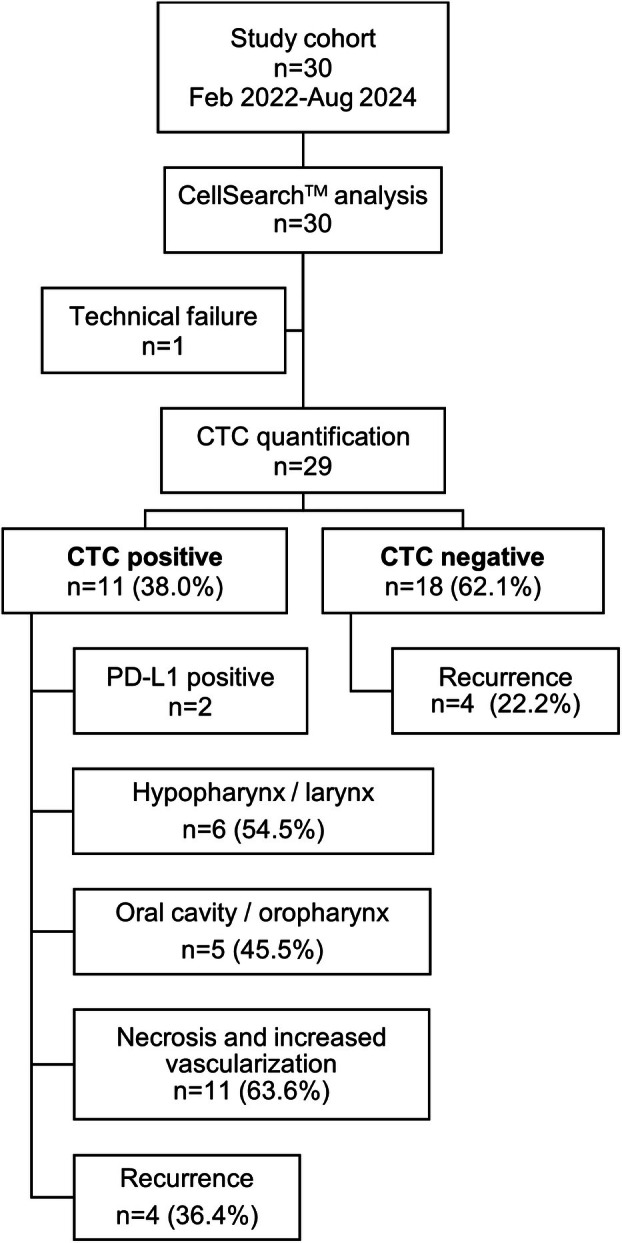
Flow chart of the study design showing the distribution of HNSCC patients with respect to CTC status.

There was no preference in terms of primary tumor localization and detection of CTC: 6/11 (54.5%) patients had hypopharyngeal or laryngeal cancers, respectively, and 5/11 (45.5%) patients had oral cavity or oropharyngeal cancers. CTCs were detectable at all disease stages, including stage I disease. Radiological analysis showed tumor necrosis and increased vascularization in 7/11 (63.6%) cases with baseline CTC detection. Median tumor volume in CTC‐positive cases was 5200.70 mm^3^ (range 1600.00–10170.00 mm^3^). No CTC clusters were detected (Fig. 1).

### 3.3 Correlation of CTC and prognosis

**Fig. 2:**
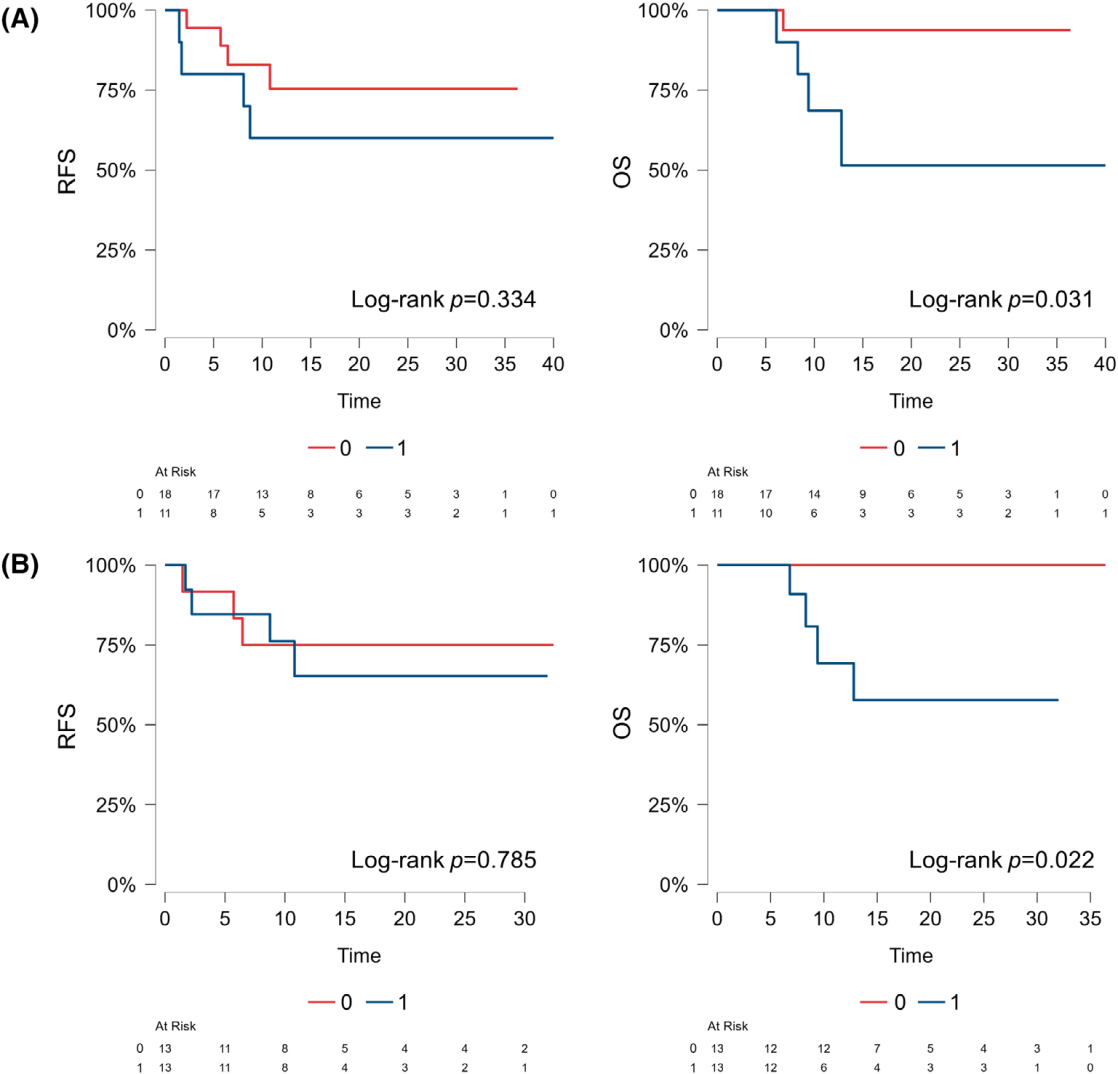
Survival analysis. Kaplan–Meier survival curves were generated for patients stratified by (A) presence or absence of circulating tumor cells (CTC) at baseline (RFS, *P* = 0.334; OS, *P* = 0.031) and (B) tumor volume (higher or lower the median) (RFS, *P* = 0.785; OS, *P* = 0.022). The log‐rank test was used to compare survival between groups. The number of patients at risk at each time point is shown below the Kaplan–Meier plots.

**Fig. 3:**
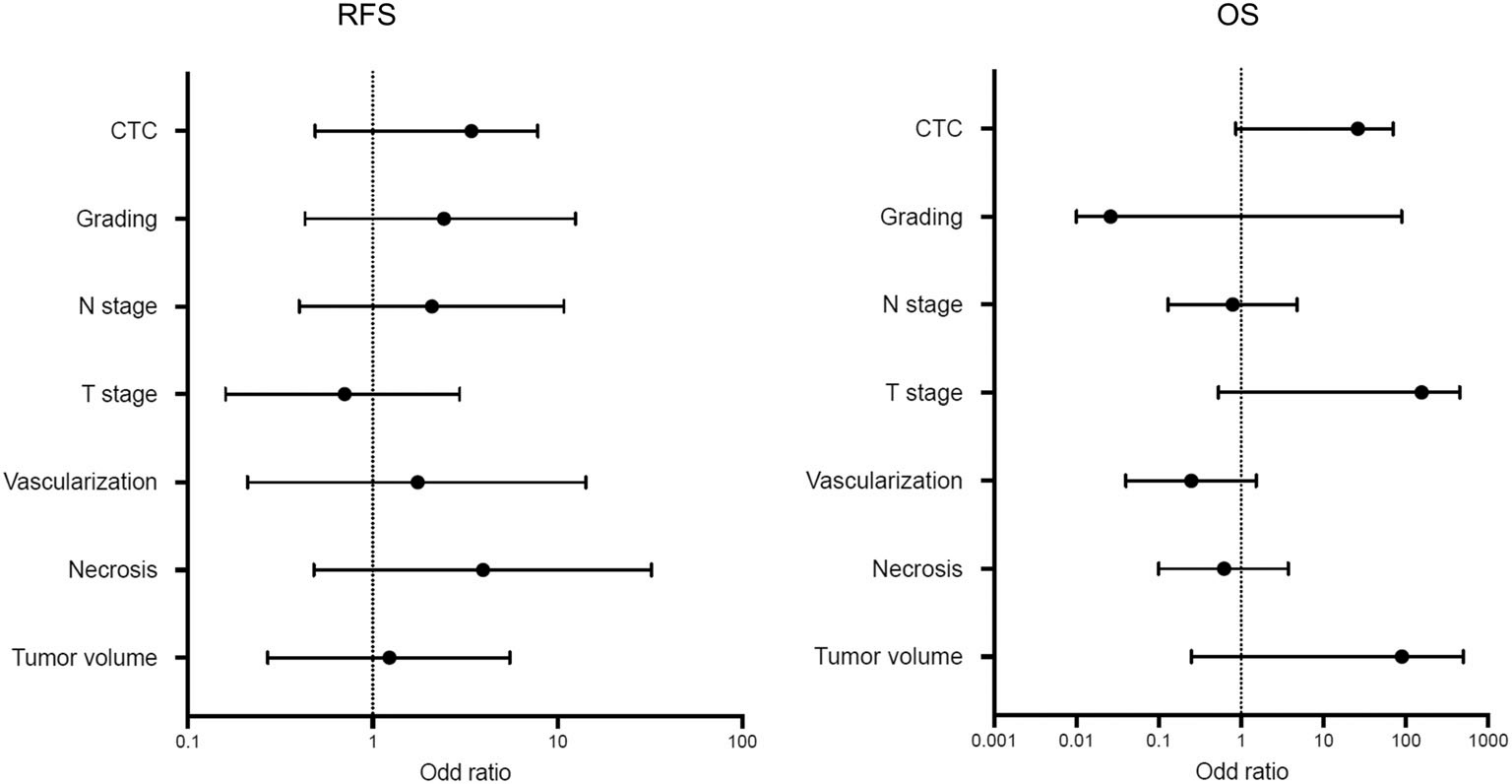
Univariate Cox regression of potential prognostic factors for recurrence‐free survival and overall survival. The hazard ratios (HRs) and 95% confidence intervals (CIs) for each parameter were calculated using univariate Cox proportional hazards regression.

The association between baseline CTC and patient clinical outcome was evaluated by analyzing its relationship with risk of relapse and survival endpoints. The cohort had a median RFS of 12.2 months and a median OS of 12.8 months (range: 0.2–40 months). Radiological parameters such as tumor volume, pT stage, pN stage, tumor necrosis and vascularization as well as presence of CTCs at baseline were included in the analysis. Kaplan–Meier survival analyses revealed that the presence of CTCs (*P* = 0.031) (Fig. 2A) and tumor volume (*P* = 0.022) (Fig. 2B) were prognostic for shorter OS. None of the other parameters showed a significant association with RFS or OS (all *P* > 0.05, data not shown). Univariate Cox regression analysis showed a trend toward an association between OS and presence of CTCs (HR: 7.75 [95% CI: 0.86–69.92], *P* = 0.068; bootstrap estimate: *P* = 0.023) and tumor volume (HR: 89.707 [95% CI: 0.25–3.2 × 10^5^], *P* = 0.281; bootstrap estimate: *P* = 0.001) (Fig. 3 and Table 2). Bootstrap resampling suggests that this association was relatively stable across iterations; however, given the small size cohort, the limited number of events and the different methods of analysis, these findings should be interpreted with caution.

**Table 2:** Univariate Cox regression of potential prognostic factors for recurrence‐free survival and overall survival. Significant *P*‐values are indicated in bold. Internal validation was performed by bootstrap resampling (1000 iterations) to assess the stability and robustness of estimates (significant values are indicated in bold under P*).

	Recurrence‐free survival				Overall survival				
	Log‐rank	Univariate analysis			Log‐rank	Univariate analysis			P*
	*P*	HR	95% CI	*P*	*P*	HR	95% CI	*P*	
CTC	0.334	1.956	0.488–7.83	0.343	**0.031**	7.757	0.86–69.92	0.068	**0.023**
Volume	0.785	1.232	0.27–5.51	0.785	**0.022**	89.707	0.25–3.2 × 10^5^	0.281	**0.001**
Necrosis	0.166	3.952	0.48–32.21	0.199	0.600	0.621	0.10–3.73	0.603	0.589
Vascularization	0.596	1.751	0.21–14.24	0.600	0.105	0.249	0.04–1.52	0.133	**0.023**
T stage	0.631	0.705	0.16–2.95	0.633	**0.002**	155.53	0.53–4.5 × 10^5^	0.216	**0.001**
N stage	0.369	2.090	0.40–10.82	0.380	0.797	0.790	0.13–4.77	0.797	0.806
Grading	0.294	2.432	0.43–12.50	0.310	0.136	0.026	0.00–89.51	0.381	**0.001**

### 3.4 Integration between CTC status and radiological parameters

**Fig. 4:**
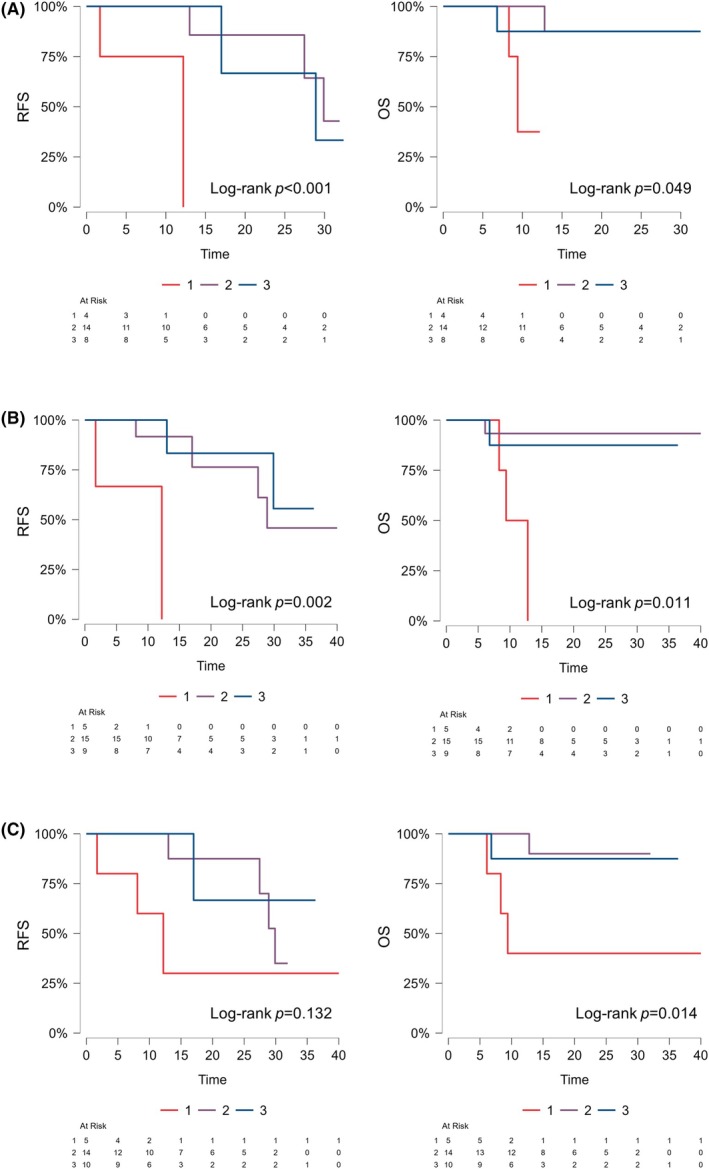
Survival analysis based on the integration of CTC with radiological parameters. Kaplan–Meier survival curves were generated after stratifying patients according to the presence or absence of circulating tumor cells (CTC) and (A) tumor volume (RFS, *P* < 0.001; OS, *P* = 0.049), (B) pathological nodal status (RFS, *P* = 0.002; OS, *P* = 0.011), and (C) pathological tumor stage (RFS, *P* = 0.132; OS, *P* = 0.014). Red curves represent patients with detectable CTC and a high value of the corresponding parameter (Group 1, high risk); purple curves represent patients without detectable CTC and a high value of the corresponding parameter (Group 2, medium risk); blue curves represent patients without detectable CTC and a low value of the corresponding parameter (Group 3, low risk). Differences between survival curves were assessed using the log‐rank test. The number of patients at risk at each time point is shown below the Kaplan–Meier plots.

Following the assessment of the individual prognostic significance of CTCs and radiological variables, a subsequent analysis investigated whether combining these factors may enhance RFS and/or OS prediction. To facilitate this integrative evaluation, CTC status was paired with each radiological or pathological parameter to stratify the cohort into three risk categories: a high‐risk group defined by the presence of detectable CTCs and a high value for the second parameter; a medium‐risk group comprising the absence of detectable CTCs alongside a high value of the second parameter; and a low‐risk group, characterized by both the absence of detectable CTCs and a low value of the second parameter. Log‐rank analysis showed that integrating CTC status with either tumor volume (Fig. 4A) or pathological nodal status (pN) (Fig. 4B) revealed statistically significant associations with both RFS (*P* < 0.001 and *P* = 0.002, respectively) and OS (*P* = 0.049 and *P* = 0.011, respectively), while integrating CTC status with pathological tumor stage (pT) (Fig. 4C) showed a statistically significant association only with OS (*P* = 0.014). Patients classified as high‐risk had a significantly greater likelihood of relapse and a shorter OS compared with those in the low‐risk group.

## 4 Discussion

HNSCC represents a group of aggressive epithelial malignancies that are associated with a poor prognosis, particularly in advanced stages. Even with multimodal therapies such as surgery and chemoradiotherapy, the 5‐year survival rate remains low. A major clinical challenge in HNSCC is the absence of reliable biomarkers to enable early detection, accurate risk stratification, and personalized treatment planning. Against this backdrop of limited and methodologically variable biomarker approaches, liquid biopsy has emerged as a promising, minimally invasive strategy to capture tumor‐derived components in real time, offering new opportunities for early detection, disease monitoring, and personalized management in HNSCC. Among the various liquid biopsy analytes, CTCs are of particular interest, as they provide intact, viable tumor cells that can be characterized to yield both phenotypic and functional insights into tumor progression and metastatic potential. Prior HNSCC studies have evaluated CTCs using a range of technologies, including CellSearch, RT‐PCR‐based assays, negative depletion/immunocytochemistry, flow cytometry, and multiplex phenotypic assays. Using CellSearch, Bozec et al. detected CTCs in 16% of patients with locally advanced HNSCC prior to treatment [17], whereas Nichols et al. reported a positivity rate of 40% in stage III/IV HNSCC [19]. Buglione et al. observed relatively low detection rates but suggested that longitudinal CTC dynamics may correlate with treatment response [24], and Tinhofer et al. demonstrated that postoperative CTC positivity was associated with inferior RFS and OS in non‐oropharyngeal squamous cell carcinoma [25]. In contrast, studies using non‐CellSearch‐based approaches [2, 26, 27] have generally reported higher detection rates and, in some cohorts, stronger associations with nodal disease, recurrence risk, and survival outcomes, underscoring the impact of assay design on both sensitivity and biological interpretation. More recent prospective data further support the clinical relevance of dynamic CTC assessment. In the CIRCUTEC study, a multicenter prospective trial in recurrent or metastatic HNSCC, CTC detection rates varied substantially by platform (69% with EPISPOT vs. 21% with CellSearch, and 11% with flow cytometry), highlighting marked assay‐dependent sensitivity [28]. Importantly, early CTC kinetics (day 0 to day 7), particularly when assessed by EPISPOT epidermal growth factor receptor (EGFR), were significantly associated with progression‐free survival, supporting the utility of CTCs as a real‐time biomarker of treatment response. In this study, preoperative CTC detection, observed in more than one‐third of patients, was associated with poorer OS, suggesting potential prognostic relevance in this cohort. CTC counts did not differ significantly between early‐stage (I–II) and advanced‐stage (III–IV) patients, consistent with previous results obtained with ctDNA [29]. This finding suggests that tumor burden alone does not fully account for shedding dynamics and that intrinsic biological factors, such as proliferation, may contribute. Furthermore, the detection of CTCs in early‐stage disease remains challenging due to their low abundance, potentially compounded by the limited blood volumes analyzed within clinically acceptable constraints. Previous studies indicate that increasing the analyzed blood volume, such as through leukapheresis, can substantially improve CTC detection rates across multiple cancer types [30, 31, 32, 33, 34]. Accordingly, future studies in HNSCC should explore strategies such as leukapheresis or serial sampling to enhance detection sensitivity. On the contrary, when combined with tumor volume or nodal stage, CTC counts enhanced risk stratification and identified a high‐risk subgroup with significantly worse survival outcomes. The integration of imaging biomarkers with liquid biopsy parameters such as CTCs represents a promising strategy to refine risk stratification and guide personalized management of HNSCC. While conventional radiological assessment provides essential information on tumor extent, morphology, and nodal status, it primarily captures static anatomical features. In contrast, liquid biopsy complements this by reflecting tumor biology and systemic dissemination potential in real time. As demonstrated in our cohort, baseline CTC positivity adds complementary value to established imaging criteria, and their combined analysis improves the identification of high‐risk patients with aggressive disease who may benefit from intensified perioperative monitoring or adjuvant therapy. CTCs were detected in 37.9% of patients, aligning with published detection rates for HNSCC and confirming that these rare cells can be reliably identified even at early‐stage disease. Importantly, CTC positivity alone emerged as a prognostic indicator for shorter survival, although univariate Cox regression did not reach statistical significance in all endpoints, likely reflecting the limited sample size of this pilot cohort. Integration of CTC status with tumor volume, pathological nodal status or tumor stage appeared to improve risk stratification in this cohort, suggesting potential clinical utility of combining liquid biopsy metrics with conventional radiological assessment. However, no direct correlation was observed between presence of CTCs and tumor volume. Patients who were CTC‐positive at baseline and exhibited a larger tumor volume or an advanced nodal stage constituted a high‐risk group characterized by reduced survival and higher relapse rates. These synergistic effects are clinically relevant, supporting more nuanced preoperative risk stratification than reliance on imaging or pathological staging alone. Radiologically detected tumor necrosis and vascularization were also more common in CTC‐positive patients, further supporting the hypothesis that aggressive tumor biology drives the release of CTCs into the circulation. This observation aligns with preclinical work linking CTC release to invasive subclones with enhanced metastatic capacity and vascular remodeling [13, 17, 18, 26, 28, 35, 36, 37]. Comparisons with previous studies confirm the broad prognostic value of CTCs across solid tumors but also underscore the challenges of translating liquid biopsy approaches into routine management of HNSCC. Recent studies have shown that radiomic and volumetric parameters correlate with tumor hypoxia vascularization, and immune microenvironment [38, 39, 40], features that may also influence CTC release and metastatic competence [11]. The combined assessment of CTCs and imaging‐derived markers provides a more comprehensive characterization of tumor behavior, bridging morphological and molecular phenotyping. Incorporating both modalities into clinical workflows could facilitate dynamic treatment adjustments, enhance early relapse detection, and guide patient selection for precision‐targeted therapies. Our findings suggest clinically relevant implications. Preoperative detection of CTCs may enable risk‐adapted treatment strategies by identifying patients with biologically aggressive disease that is not fully captured by conventional staging. Notably, the integration of CTC status with imaging‐derived parameters (particularly tumor volume and nodal stage) substantially improved risk stratification compared to either modality alone. In this context, CTC‐positive patients with adverse radiological features may represent a high‐risk subgroup that could benefit from intensified perioperative management, including closer surveillance, earlier initiation of adjuvant therapy, or prioritization for clinical trials investigating novel systemic or immunotherapeutic approaches. Moreover, the combined use of liquid biopsy and radiological assessment offers complementary insights into tumor biology and morphology, capturing both systemic dissemination and local tumor characteristics. This multimodal approach has the potential to enhance preoperative decision‐making and refine patient selection for personalized treatment strategies. While these findings require validation in larger prospective cohorts, they support the clinical feasibility and added value of integrating CTC analysis with imaging into routine preoperative risk assessment in HNSCC. In addition to enumeration, we assessed PD‐L1 expression on CTCs as a potential biomarker. PD‐L1 positivity was detected in a small subset of patients with CTCs, none of whom developed disease recurrence during follow‐up. The absence of recurrence contrasts with some prior reports [41] and likely reflects the limited sample size and low event rate. Therefore, these findings should be interpreted with caution. Emerging evidence in HNSCC suggests that PD‐L1 expression on CTCs may carry both biological and clinical significance, reflecting dynamic immune evasion mechanisms. In this context, PD‐L1–positive CTCs have been proposed as a minimally invasive biomarker to guide patient selection for immune checkpoint inhibitors and to enable real‐time monitoring of treatment response [42, 43]. This may be particularly relevant in HNSCC, where PD‐1/PD‐L1 targeted therapies are increasingly being integrated into earlier disease stages, including the neoadjuvant setting, as demonstrated in trials such as KEYNOTE‐689 [44]. In this evolving therapeutic landscape, minimally invasive biomarkers are urgently needed to optimize patient selection and to better understand treatment response. Assessment of PD‐L1 expression on CTCs could complement tissue‐based testing by capturing spatial and temporal heterogeneity and may enable real‐time monitoring of immune‐related tumor dynamics during neoadjuvant treatment. Furthermore, CTC‐based PD‐L1 analysis may help identify patients more likely to benefit from neoadjuvant immunotherapy or, conversely, those who may require alternative or intensified treatment strategies. However, the predictive value of PD‐L1–positive CTCs in this setting remains to be established. Prospective studies incorporating serial liquid biopsy sampling alongside immunotherapy trials are warranted to determine whether this approach can improve patient stratification and ultimately clinical outcomes. Overall, while PD‐L1 expression on CTCs represents a promising biomarker in HNSCC, its prognostic and predictive value remains insufficiently defined and warrants further investigation in larger, well‐controlled prospective studies. Several limitations of this study should be acknowledged. Most notably, the relatively small cohort size, limited number of clinical events, and short follow‐up period restrict the statistical robustness of survival analyses. Therefore, the reported associations should be interpreted with caution and considered exploratory. An additional methodological consideration relates to the discrepancy observed between conventional Cox regression and bootstrap‐adjusted results. In several instances, statistical significance was observed only after bootstrap resampling. It is important to note that bootstrap analysis in this study was primarily applied as an internal validation tool to assess the stability of the identified associations, rather than to establish statistical significance. The observed differences likely reflect the limited sample size and small number of events, which can lead to instability of *P*‐values in conventional models. Therefore, bootstrap‐derived estimates should be interpreted as supportive of potential robustness, but not as definitive evidence, and the overall findings should be considered exploratory.

## 5 Conclusion

CTCs detected via liquid biopsy may provide dynamic insight into tumor biology that conventional imaging alone cannot capture, addressing a critical unmet need in HNSCC management. Our data suggest integrating baseline CTC detection with imaging‐derived tumor characteristics to refine preoperative risk stratification in surgically treated HNSCC patients. This multimodal approach could lay the foundation for more tailored perioperative interventions and follow‐up algorithms, ultimately improving outcomes in a population at high risk for recurrence and mortality. Larger prospective studies are warranted to validate these findings and facilitate the translation of liquid biopsy into routine clinical practice.

## Conflict of interest

The authors declare no conflict of interest.

## Author contributions

SF and MA‐F conceived and designed the study. SF, TH, AL, LK, and MA‐F acquired and analyzed the data. MA‐F performed the statistical analyses. SS and GA provided analysis of the radiological data. PB, MC, and JR supervised the study, contributed to data interpretation, and provided critical revision of the manuscript for important intellectual content. All authors contributed to data interpretation as well as development, writing and approval of the paper.

## Data Availability

The datasets used and analyzed during the current study are available from the corresponding author on reasonable request.
